# Erratum: A reference bacterial genome dataset generated on the MinION^TM^ portable single-molecule nanopore sequencer

**DOI:** 10.1186/s13742-015-0043-z

**Published:** 2015-02-13

**Authors:** Joshua Quick, Aaron R Quinlan, Nicholas J Loman

**Affiliations:** 1 Institute of Microbiology and Infection, University of Birmingham, Birmingham, B15 2TT UK; 2 NIHR Surgical Reconstruction and Microbiology Research Centre, University of Birmingham, Birmingham, B15 2TT UK; 3 Center for Public Health Genomics, University of Virginia, VirginiaUS, VA 22908 Charlottesville,

Recently we noticed that we could not reproduce Figure three (Figure 1 here) while analysing this data as part of a new experiment [1]. This was due to a modification to the script used for extracting sequence alignment profiles from the dataset [2]. On further investigation we found that an error in this script had been reported and a fix supplied by an anonymous online contributor on October 27th 2014 via Github [3]. The error prevented mismatches from being properly counted in the calculation of read accuracy (insertions and deletions were). We therefore present updated versions of Figures three (Figure 1 here) and four (Figure 2 here) generated by the corrected script. We are grateful to the anonymous contributor who noticed this error. The manuscript text and tables are unaffected.

**Figure 1:**
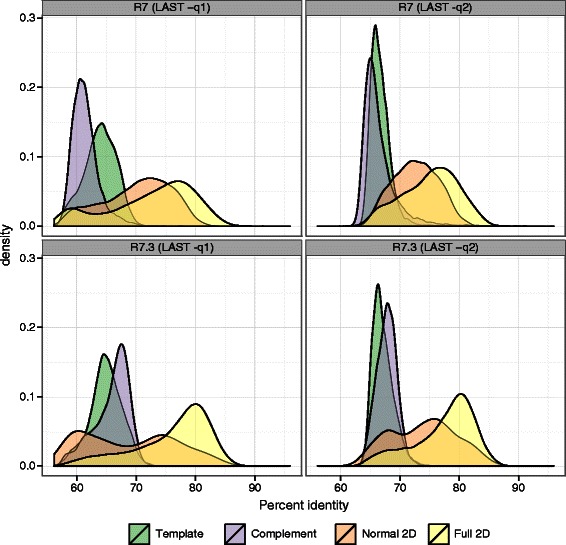
**Kernel density plots showing accuracy for R7 and R7.3 chemistries with two different values for the LAST substitution penalty score.**

**Figure 2:**
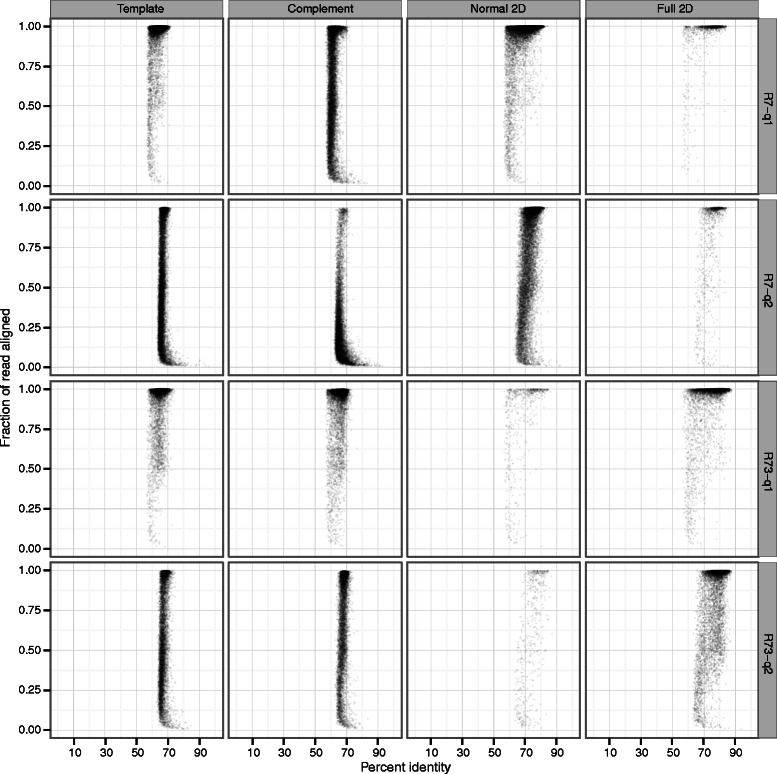
**Alignment identity and completeness.** Each plot reflects the alignment identity and the proportion of the read aligned for all 2D reads, as well as the underlying template and complement sequences. The top two panels reflect the alignment results for normal and full 2D reads from the R7 flowcell, and the bottom two panels reflect the R7.3 flowcell. Left panels employ a mismatch penalty of 1 and right panels reflect a mismatch penalty of 2. Overall, the lower mismatch penalty increases the identity and fraction of the read that aligned and this effect is greatest for full 2D reads.

We would also like to correct the spelling of Minh Duc Cao in the Acknowledgements section.

We apologise for any inconvenience caused by this error.
